# One Health: Addressing Global Challenges at the Nexus of Human, Animal, and Environmental Health

**DOI:** 10.1371/journal.ppat.1005731

**Published:** 2016-09-15

**Authors:** Waithaka Mwangi, Paul de Figueiredo, Michael F. Criscitiello

**Affiliations:** 1 Department of Veterinary Pathobiology, College of Veterinary Medicine, Texas A&M University, College Station, Texas, United States of America; 2 Department of Microbial Pathogenesis and Immunology, College of Medicine, Texas A&M Health Science Center, Bryan, Texas, United States of America; 3 Norman Borlaug Center, Texas A&M University, College Station, Texas, United States of America; 4 Comparative Immunogenetics Laboratory, Texas A&M University, College Station, Texas, United States of America; University of Pittsburgh, UNITED STATES

## What is One Health?

It is estimated that 60% of recently emerging human diseases, including HIV-1 and pandemic influenza, originate from animals [1–4]. The increasing pressures of zoonoses, which are infectious diseases of animals that can be naturally transmitted to humans, have their roots in many causes. MacCready has estimated that since the dawn of human agriculture, the terrestrial vertebrate biomass has shifted from humans and their domesticated species accounting for ~0.1% 10,000 years ago to 98% today [5]. These approximations demonstrate the exponential growth of opportunities for pathogens to spread with increasing ease from the animals upon which we depend to us. Besides growth of human and animal populations, many other factors drive zoonoses. These include habitat destruction and the resultant increased contact between humans and wildlife; bushmeat consumption, which was linked to HIV-1 infections in humans [2], and climate change, which influences the geographic range of many disease vectors. Range expansion into areas heavily populated by humans and human encroachment into the habitats of animal reservoirs also increase the risk of human infection [6]. The global economy has enabled the rapid spread of people, animals, plants, and agricultural products across the world. This mobility has contributed to more frequent outbreaks of zoonotic diseases and infections of naive populations [7]. To address these diverse challenges, innovative ways of thinking about health from an integrated perspective that countenances human, animal, and environmental factors must be developed.

Since ancient times, our understanding of human medicine has been informed by the study of animals. In fact, until the early 20th century, the studies of human and animal medicine were closely intertwined [8]. However, with the drive for specialization that accompanied the Industrial Revolution, linkages between the practice of human and animal medicine decreased. Veterinary and human medicine became distinct disciplines with separate training, compliance, funding, and professional societies, and interactions between professionals with training in these disciplines declined. Recently, there has been a renewed interest in re-bridging these now disparate fields, as well as incorporating the environmental sciences, under the heading of “One Health,” which is defined as a cross-disciplinary initiative to consider diverse data and interdependencies in managing human, animal, and environmental well-being [9,10].

Several features of this definition of One Health are notable. The definition is broad and encompasses both conceptual and operational aspects. Conceptually, the definition demands that analyses conducted under the auspices of the “One Health” moniker include a consideration of environmental factors. Operationally, One Health encompasses a worldwide strategy for expanding interdisciplinary collaboration and communication in germane aspects of human, animal, and environmental health. The Ebola epidemic of 2013–2015 serves as a case study in which the One Health initiative can be examined.

## The West African Ebola Outbreak of 2013–2015

The Ebola outbreak of 2013–2015, which started in Guinea and raised the specter of pandemic spread [11], provides a useful lens through which to analyze the One Health approach. Previous Ebola outbreaks were self-limiting due, in part, to the fact that they occurred in remote regions [12]. However, the scale and the spread of the recent outbreak were unprecedented. The infection threatened health providers in international gateway cities, and contingencies for satellite spread burdened global health care infrastructure [13]. In fact, health care systems in the affected regions in West Africa were rendered dysfunctional to the extent that other common diseases, such as malaria, caused additional deaths, and it was suggested that this added mortality exceeded the outbreak itself [14]. Analysis of real-time responses to the outbreak highlight many challenges in modeling not only the environmental facilitation, zoonosis, and human spread of Ebola but also the related dynamics of other disease (e.g., through interruption of vaccination programs) [15]. Economic activities also slowed down or came to a halt, thereby negatively impacting the livelihood of residents of affected regions [16]. This “perfect storm” [17] was made possible by several interconnected factors that were responsible for not only the initial outbreak in humans but also the rapid spread of the virus from the epicenter.

**Fig 1:**
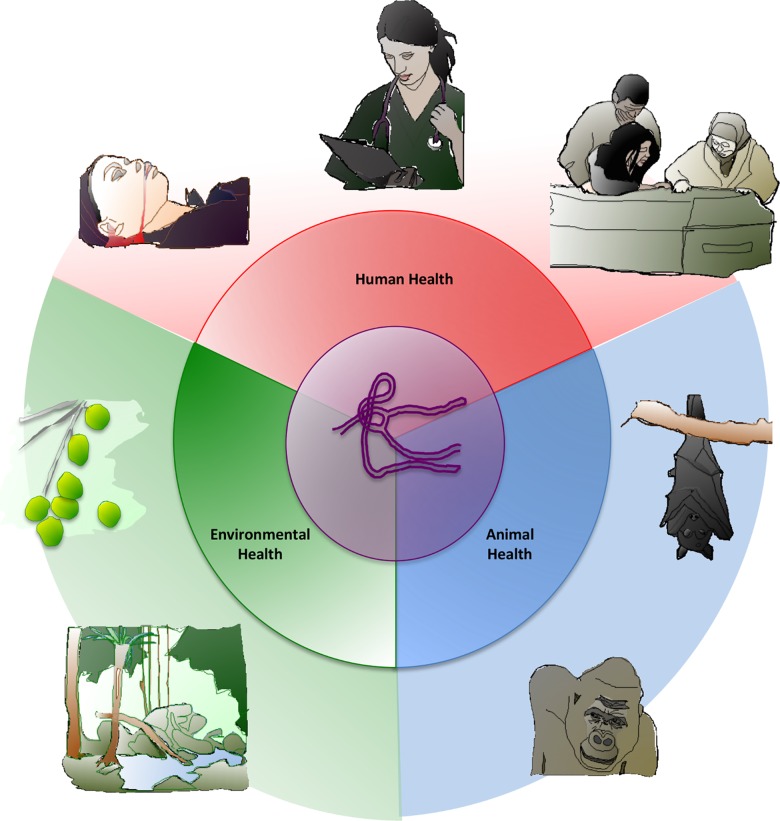
The One Health concept and components. The One Health concept allows for an emerging infectious disease such as the Ebola epidemic to be viewed and addressed in a tripartite manner: factors from human (pink), animal (blue) [52], and environmental (green) health are considered in the forecast and control of disease.

The human-to-human route of Ebola infection became common knowledge during the global alarm of the recent outbreak. Less appreciated was the zoonotic origin of the virus: current data point to several species of fruit bats as the natural reservoirs of the pathogen (**Fig 1**) [18,19]. Human exposure to bat populations is usually limited to consumption as a privileged delicacy, but periods of economic stress forced more people to expand food options to include potentially infected bats [20]. Similarly, poverty encouraged foraging deeper into more remote, bat-laden forests in search of bushmeat, fruits and seeds, and edible plants. Bushmeat from non-human primates is a potential source of human infection, as butchering and consumption of these animals was implicated in Ebola outbreaks during the 1990s in the Congo basin and in Gabon [12]. The behavioral ecology and biogeography of bats are not static. Deforestation, length of the dry season, and global warming influence the territories bats must occupy to access fruit and mates. Bat migration is a potential source for virus spread among bat populations as well as a source of greater interactions with humans [21], and the very activity of flight has been implicated in selecting for viral symbionts of bats that are adapted to high metabolism and febrile daily cycles in these mammals [22]. The 2013–2015 outbreak focused much attention on the mapping of human–human contacts in the early stages of the epidemic [23] but less to the bat–human contacts (with possible intermediates such as cave floor guano or infected bushmeat) and less still to the animal health and environmental factors that may have influenced these interactions. These are the areas where a One Health approach would prove informative.

**Fig 2:**
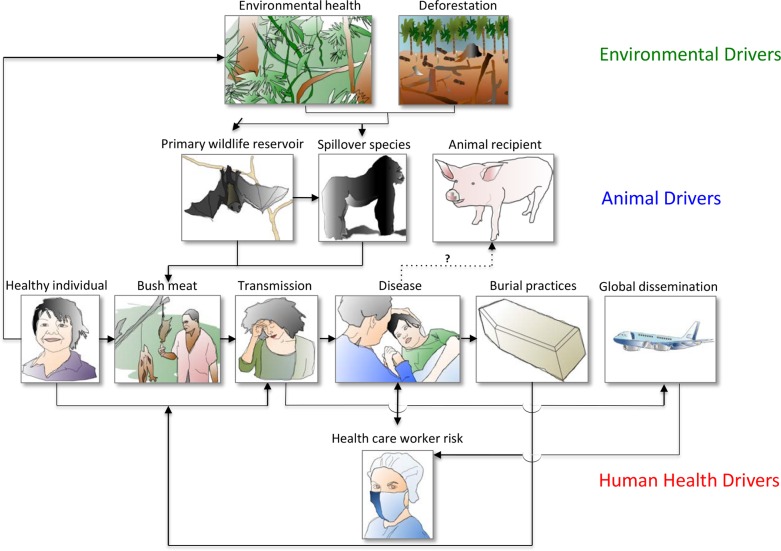
Ebola as a case study of interactions between human, animal, and environmental health drivers. Climactic and anthropomorphic factors influence environmental drivers such as drought and deforestation. These factors in turn affect the populations and migrations of the primary reservoir of the Ebola virus, fruit bats, as well as other animals that may prey upon them or compete with them for fruit. These animal drivers influence the routes and rates of human infection through bushmeat consumption and possibly other means. Human-to-human spread is exacerbated by burial practices, caregiver barrier protocols, and global travel. Human drivers of disease feed back to influence animal health via possible pet infection and the transport of infected wildlife, and environmental health through increasing human–animal contacts through harvesting natural resources from remote forests.

Unlike previous incidences, the West African outbreak became a global threat due, in part, to increased spread by humans (**Fig 2**). The initial outbreak in Guinea rapidly disseminated regionally, mainly due to virus transmission by relatives from neighboring countries who were exposed during burial rites. Unlike previous outbreaks that occurred in remote areas, transport infrastructure promoted regional and international human-to-human virus dissemination [24]. The situation was made worse by slow responses to quarantine infected patients and quickly spread because of poor health care infrastructure and non-compliance with requests for quarantine and other control methods [17]. The implementation of well-planned One Health approaches that bring together all stakeholders to address the interconnected factors responsible for enabling a pathogen such as Ebola to spread so successfully, including cultural practices, environmental conditions, and wildlife dynamics, can help address future Ebola epidemics.

## What Are the Gaps That Would Be Addressed by a One Health Approach to a Future Outbreak?

The domestic and international response to the recent Ebola crisis was complex and multimodal. It included the implementation of public health, public awareness, and clinical intervention measures [25]. As a result of these measures, the outbreak was eventually contained. However, the crisis revealed several significant gaps in disease awareness and management that can be addressed in responses to future outbreaks of emerging diseases. These gaps include the following: (1) insufficient monitoring and ecological modeling of zoonotic infection and transmission, (2) insufficient systems for rapid dissemination of and community education about the ecological aspects of disease outbreak and management, and (3) insufficient resources committed to enhancing food security to limit environmental encroachment and exposure to zoonotic disease in the wild.

## How Will These Gaps Be Filled?

First, it is not clear exactly how humans were initially exposed to Ebola, but available information suggests that it could have been through handling infected fruit bats [26]. Despite increased knowledge of cultural practices and bat behavior in Ebola endemic regions, more studies are needed to generate the data required to create computational models that will accurately predict outbreaks [27,28]. Evidence of asymptomatic infection in fruit bats suggests that these mammals may be natural Ebola reservoirs [26], but there is a need to empirically identify ecological drivers of virus spillover and how these drivers influence infection of other susceptible hosts, including humans. Recently, the virus has been discovered to reside in immune-privileged organs (e.g., eyes and testes) several months after initial infection [29,30]. This raises questions about how long infectious forms of the virus can be maintained in infected hosts and if there are factors that promote latency. In addition, it is important to determine whether Ebola causes acute or chronic infection in natural reservoirs and whether certain factors, such as stress, can lead to high viral loads that are shed in wastes or contaminate the environment, including wild fruits on which bats and other wildlife feed [31]. Such pulses have been found in Marburg, Nipah, and Hendra shedding from bats [32–34]. Furthermore, seasonal monitoring of Ebola virus in wildlife will reveal the temporal profile of viral load in these hosts and these data will reveal when there is an increased probability of zoonotic infection and transmission.

Second, to address deficiencies in information dissemination and community education about the ecological aspects of Ebola outbreaks, a coordinated response is required [35–37]. Studies have revealed striking deficiencies in community understanding of modes and ecological drivers of Ebola transmission as well as causes of and effective interventions for Ebola Virus Disease (EVD) [38,39]. Community members also demonstrate limited understanding of the zoonotic origin of the pathogen and the risks associated with exposure to wildlife reservoirs, including fruit bat populations [26]. To address these deficiencies, investments in health education campaigns that incorporate ecological dimensions and are sensitive to regional challenges, including limited resources to support public health professionals and low literacy rates, must be pursued. Previously, educational programs implemented in partnership with local schools and churches were successful [40]. The dissemination of relevant disease ecology information through leaflets, posters, and banners was also employed. For such campaigns to be successful, they must display cultural competence and respect for local communities and their leaders as well as develop trust between primary educators and the larger populace [38]. Resources provided by bilateral partners, non-governmental organizations, and local governments are essential to implement the requisite educational activities [41], including those that incorporate One Health components.

Third, resources must be committed to ensure that unstable food security does not increase opportunities for humans to encounter viral reservoirs. Hunger drives humans into more remote forests where alternative food sources, including primate bushmeat, are available. Although bat saliva and feces have been implicated in Ebola virus transmission to primates [42,43], predation of bats by monkeys has also been documented [44] and could contribute to non-human primate infection. Harvesting of bushmeat has been shown to be in inverse proportion to fisheries production [45]. Both fish and bushmeat have been identified as dietary substitutes in Ghana [46]. Thus, changes in the frequencies of human encounters with viral wildlife reservoirs can be better predicted and managed with attention to such seemingly discrete parameters as marine resource management and the El Niño global oscillation. However, even if nutritional abundance can be ensured, local culture will likely maintain some significant demand for bushmeat, as unsustainable consumption of wildlife remains a problem even in relatively prosperous countries [47]. As meteorological, regulatory, and economic conditions contributing to food security crises are often predictable [48], resource allocation for a forewarned humanitarian response can mitigate the need for environmental encroachment and wildlife harvesting.

There are programs in place that are monitoring disease burden in wildlife species, including the United States Agency for International Development (USAID) Emerging Pandemic Threats program. Despite this program’s successful monitoring of pathogens in wild reservoirs, lack of coordination with other entities such as the World Organization for Animal Health (OIE) and CDC has prevented better coverage of disease outbreaks. The consequences of such One Health approaches to these insufficiencies are more robust data informed by human public health professionals, veterinary wildlife pathologists, and environmental scientists for monitoring outbreak potential and responding more nimbly.

## What Is the Way Forward for the One Health Paradigm?

The implications of implementing the One Health vision are manifold, especially in the developing world, where the impact of zoonotic and emerging diseases is most acutely experienced. For example, human, animal, and environmental health are essential for long-term economic prosperity, reduction in foreign aid dependency, and political stability. In terms of impact on the community of researchers investigating host–pathogen interactions, the One Health approach implies a recalibration of training to address the relative paucity of investigators prepared to address the threat of global zoonotic infections, including hemorrhagic diseases, in which proficiency working at high biocontainment levels needs to be cross-trained with a One Health consideration of animal and environmental factors. Funding agencies must prioritize host–pathogen interactions as well as the broader health of reservoir and vector species and the environments containing them.

Despite these challenges, there are several recent advances that provide optimism. Early results suggest that several experimental biopharmaceutical therapeutics, such as ZMapp, are effective against the virus [49]. Numerous prototype vaccine candidates are also being fast-tracked for field testing, and preliminary results from non-human primate studies suggest that these prototypes have potential to be effective prophylactics [50]. Regarding manpower and capacity building, medical schools, veterinary colleges, and research universities are beginning to realize the need for training students and professionals in One Health, and several programs with Global Health or One Health foci have emerged [51]. Understanding and integrating environmental and animal health factors germane to One Health in general, and the Ebola outbreak in particular, are also needed to fully address the Ebola challenge and future scourges.

We must remain cognizant that human well-being is intimately tied to the health of domestic and wild animals and the environment. As human populations expand, interactions between these three sectors will continue to increase. Specialists in environmental sciences, veterinary medicine, and human medicine need to help each other in the stewardship of a biosphere capable of maintaining a healthy human populace, the animals it depends upon, and the environment to sustain all.
